# Supplementary material for: Assessing Radish Health during Space Cultivation by Gene Transcription
Source: Plants (Basel). 2023 Sep 30;12(19):3458. doi: 10.3390/plants12193458 (PMC10574649; doi:10.3390/plants12193458)
Supplement: Supplementary file 1 [file plants-12-03458-s001.zip › plants-2600128-SI.pdf]

Assessing radish health during space cultivation by gene transcription.

Supplemental information:

Table S1: Select, verified primer sequences for *R. sativus* used to assess transcription data during space flight and ground control.

Accession #	Description	Primers	Sequence (5' - 3')	Start	Stop	Amplicon Length	Efficiency %
AB042186.1	myrosinase activity	F	TCAAGAACCCTGCGAAAC	1576	1594	127	101
		R	AGGAAGTGTGAGAGACATAGA	1682	1703		
AB747087.1	Chlorophyll-binding protein	F	GCAGGAGCACACACTTAC	457	475	111	89
		R	GGTCTTAGCCTCATCATTGG	548	568		
KF682449.1	Glucosinolate synthesis	F	AGCAGAAGTGAGGGAGTAT	981	1000	89	89
		R	CTAAGGCTCTGAAGTAAGGAAG	1048	1070		
EU369394.1	Peroxidase precursor	F	GGAACTCAAGGCGAGATAAG	874	894	86	94
		R	CTCACAACACCATCATCCTC	940	960		
M63843.1	Napin storage protein	F	TGAGCCGTATCTACCAGAC	423	441	64	100
		R	ATGCTAACTTGCGGGATTC	468	486		
AT5G09810.1	Actin 7	F	AGCTTCGTGTTGCACCTGAA	456	476	120	104.3
		R	ACATGGCAGGGACATTGAATG	555	576		
Z24738.1	Ubiquitin1	F	GGAGAGCAGTGACACCATCGA	81	103	120	103.8
		R	GCCAAGGTACGACCATCTTCA	180	201		

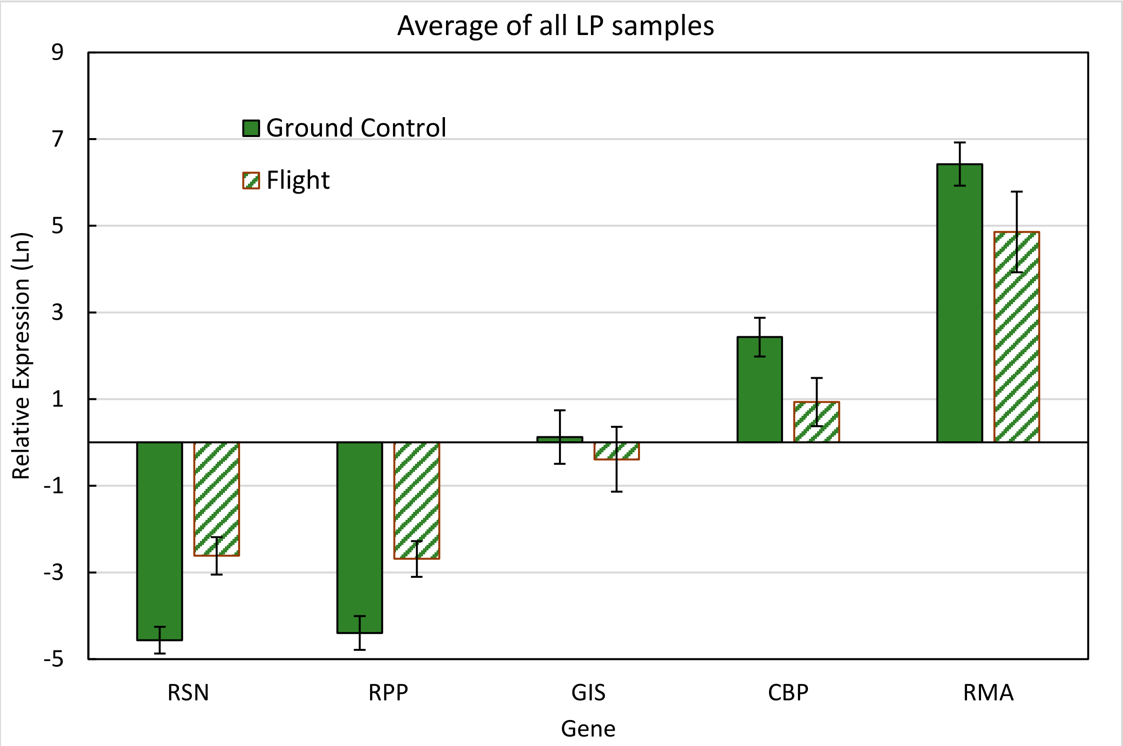

Figure S1: Average transcription of genes during two space flights and a corresponding ground control. The data represent the average of eight leaf punch samples ± SE and are shown relative to ACT7 and UBQ1 that were collected on day 10, 18 and 24 of a 27-day cultivation period of *R. sativus*, cultivar Cherry Belle.

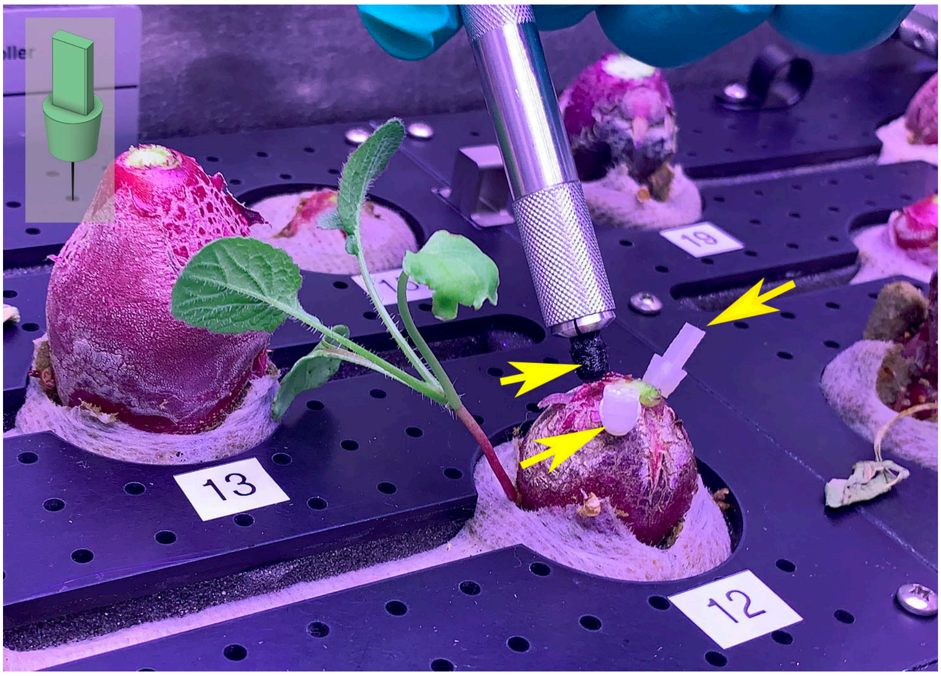

Figure S2: SPGE sampling of radish bulbs showing the holder used to insert the probes into the tissue (arrows). The insert shows a detailed sketch of the holder and probe. Sampling was done on day 27 after imbibition at the Kennedy Space Center and the second grow-out on the ISS.
